# Repeated infection by cytomegalovirus induces neuroinflammation and compromises BBB integrity

**DOI:** 10.1002/alz.093005

**Published:** 2025-01-03

**Authors:** Natasha B Barahona, Lucas P Garfinkel, Hanyun Wang, Miayla Marcus, Advaith Subramanian, Kennedy N Willis, Mark A Harrison, Sara Morris, Kevin Zwezdaryk, Elizabeth Engler‐Chiurazzi

**Affiliations:** ^1^ Tulane University, New Orleans, LA USA; ^2^ Xavier University, New Orleans, LA USA

## Abstract

**Background:**

It is well established that genetic factors are implicated in the development of Alzheimer’s disease (AD), but there is growing interest in how environmental factors like infection contribute to its progression. Recent evidence suggests that greater exposure to infections across the lifespan can potentiate the rate and severity of cognitive decline. In addition to contributing to mechanisms underlying the aggregation of Aβ fragments and phosphorylation of tau proteins, the infectious etiology of dementia may be caused by infectious agents triggering neuroinflammatory pathways and degradation of the blood‐brain barrier (BBB). To further understand neurological changes following viral infection, we investigated the effects of repeated intermittent exposure to cytomegalovirus (CMV) on neuroinflammation and BBB integrity. We hypothesized that if infection burden promotes a dementia phenotype, then mice with a higher lifetime history of intermittent viral exposure will display increased neuroinflammation and BBB permeability.

**Method:**

Beginning at 2 months of age, we exposed female Balb/c mice to murine CMV infection or mock infection every 3 months until they reached 8, 14, or 20 months of age, corresponding to 2, 4, or 6 exposures. We used immunofluorescence staining to evaluate the presence of neuroinflammatory markers GFAP and Iba‐1 and barrier permeability markers IgG, Claudin‐5, and Fibrinogen in the hippocampus and striatum, brain regions central to learning and memory function and in which dysfunction is implicated in AD.

**Result:**

Across age groups, we saw a significant increase in astrocytic activity in the hippocampus as measured by GFAP (p< 0.05). For barrier permeability markers, IgG was increased in the hippocampus across age groups (*p*<0.05), while there was a decrease in claudin‐5 (*p* = 0.0272) and an increase in fibrinogen (*p* = 0.0232) at 14 m.o. Changes in striatum were modest though we identified significant increases in Iba‐1 and fibrinogen at 14 months. Measurement of markers in 20 m.o. mice are ongoing.

**Conclusion:**

A history of intermittent CMV infection led to significant increases in reactive astrocytic and microglial activity in conjunction with compromised BBB integrity in the hippocampus. These results together provide a framework for how infection burden alters neurobiology and contributes to the progression of cognitive decline.

